# The Emerging Role of EVA1A in Different Types of Cancers

**DOI:** 10.3390/ijms23126665

**Published:** 2022-06-15

**Authors:** Huijie Zhao, Huiyang Liu, Yihan Yang, Honggang Wang

**Affiliations:** 1 Institute of Chronic Disease Risks Assessment, Henan University, Jinming Avenue, Kaifeng 475004, China; zhj5696@163.com; 2 School of Basic Medical Sciences, Henan University, Kaifeng 475004, China; m15736875597@163.com (H.L.); h1323240458@163.com (Y.Y.)

**Keywords:** EVA1A, autophagy, triple-negative breast cancer, papillary thyroid cancer, non-small cell lung cancer, hepatocellular carcinoma, glioblastoma

## Abstract

Eva-1 homolog A (EVA1A), also known as transmembrane protein 166 (TMEM166) and regulator of programmed cell death, is an endoplasmic reticulum associated protein, which can play an important role in many diseases, including a variety of cancers, by regulating autophagy/apoptosis. However, the related mechanism, especially the role of EVA1A in cancers, has not been fully understood. In this review, we summarize the recent studies on the role of EVA1A in different types of cancers, including breast cancer, papillary thyroid cancer, non-small cell lung cancer, hepatocellular carcinoma, glioblastoma and pancreatic cancer, and analyze the relevant mechanisms to provide a theoretical basis for future related research.

## 1. Introduction

Eva-1 homolog A (EVA1A; also named as transmembrane protein 166 and sequence similarity family 176), found by high-throughput screening in 2007, is a new lysosomal and endoplasmic reticulum related protein that regulates autophagy and apoptosis [1,2]. It mainly exists in humans, chimpanzees, rats, mice and dogs, suggesting that it may play an important role in vertebrates [3]. The evidence indicates that the expression of EVA1A is cell type-specific and tissue type-specific [4], and is substantially reduced in cancer tissues [4,5,6,7]. EVA1A is highly expressed in some normal cells and tissues, including the glomerular zone of the adrenal cortex, islet cells, chromophils of the pituitary gland, gastric fundus gland, squamous epithelium, esophageal mucosa and hepatocytes [4]. However, the expression of EVA1A is low in several human cancers, including esophageal and gastric cancer [4,5]. Research has shown that EVA1A can interact with Atg16L1 through its C-terminal, so as to promote the formation of the autophagosome and upregulate autophagy [7]. Other studies have shown that EVA1A can participate in the promotion of CCAAT/enhancer binding proteins alpha (C/EBPα)-mediated autophagy in hepatocellular carcinoma [8]. In addition, EVA1A can contribute to embryonic neurogenesis [9], acute liver injury [10] and cardiac remodeling [3] by regulating autophagy. It has been reported that EVA1A significantly induces the caspase-3-mediated apoptosis of cancer cells to inhibit cancer [7,11]. The latest research shows that EVA1A can also regulate HBV replication [12], suggesting that it has complex and diverse functions. More and more studies have revealed that EVA1A plays a vital role in cancers [7,13], indicating that EVA1A may provide a new strategy for the treatment of cancers. However, the relevant mechanism is not completely clear. Therefore, in this review, we review the recent progress in delineating the role of EVA1A in different kinds of cancers, and analyze the related mechanisms, hoping to provide a theoretical reference for in-depth research in the future.

## 2. The Role of EVA1A in Breast Cancer

Breast cancer is a common malignant tumor of women worldwide [14]. Triple-negative breast cancer (TNBC) refers to a specific subtype of breast cancer which expresses no expression of progesterone (PR), estrogen (ER) or human epidermal growth factor receptor-2 (HER2) [15,16]. TNBC is an aggressive tumor which accounts for nearly twenty percent of breast cancer and leads to poor clinical outcomes [17]. TNBC usually has more aggressive biological characteristics, such as early occurrence of metastatic disease, visceral metastasis, rapid disease progression, short response time to existing treatment and a low survival rate [18]. It is not sensitive to HER2 therapy or endocrine therapy, so there is no effective means to treat TNBC to date [19,20]. Flubendazole is a member of the benzimidazole family, and is widely used in clinical practice to treat worms and intestinal parasitic infections. Recent studies have shown that flubendazole may help to treat different types of cancers, such as breast cancer, by inhibiting tubulin polymerization [21]. It has been reported that EVA1A-modulated autophagy can improve breast cancer [13]. In order to further study the role and mechanism of EVA1A in TNBC, Xumin Zhou and colleagues conducted a series of experiments, and the results showed that flubendazole treatment inhibited the proliferation and colony formation of TNBC cells. Similar outcomes were acquired in a xenograft tumor model of mice in vivo. The in-depth study showed that flubendazole induced apoptosis of TNBC cells by increasing the expression levels of cleaved caspase 3 and Bax, and reducing Bcl-2 expression. Flubendazole also upregulated autophagy through increasing the levels of LC3-II/LC3-I and Beclin-1, and inducing p62 degradation. Furthermore, flubendazole induced the formation of autolysosomes and autophagosomes. However, 3-MA (an autophgy inhibitor via inhibiting autophagosome formation) inhibited autophagy and partially abolished the apoptosis induced by flubendazole, suggesting that flubendazole induced apoptosis of TNBC cells by promoting autophagy. In addition, flubendazole suppressed TNBC metastasis by decreasing MMP-2 expression and increasing E-cadherin expression in vivo and in vitro, which was relieved by 3-MA or ATG5 knockdown, indicating that flubendazole suppressed TNBC metastasis by promoting autophagy. RNA-seq analysis revealed that EVA1A expression was reduced in TNBC and upregulated in flubendazole-treated TNBC in vivo and in vitro. Silencing EVA1A with siRNA abolished the inhibition of apoptosis, autophagy and metastasis of TNBC induced by flubendazole, indicating that flubendazole upregulated autophagy and suppressed the proliferation and migration of TNBC cells through promoting EVA1A. Moreover, EVA1A overexpression could not induce the LC3 aggregation in TNBC cells with ATG5 silence, indicating that EVA1A promoted autophagy through ATG5. EVA1A overexpression also significantly inhibited the proliferation of TNBC cells, and this was abolished in ATG5-depleted TNBC cells, which further confirmed that EVA1A improved TNBC by promoting autophagy via ATG5. The above-mentioned indicate that flubendazole suppresses apoptosis and metastasis of TNBC through promoting EVA1A-induced autophagy through ATG5. The point mutation of Thr113 in EVA1A alleviated the suppression of apoptosis, autophagy and metastasis of TNBC by flubendazole, suggesting that flubendazole might promote EVA1A via binding to Thr113 of EVA1A. Summarily, EVA1A induced autophagic death of TNBC cells, resulting in suppressing the tumor proliferation and migration to improve TNBC [11]. In the above study, EVA1A promotes the autophagic death of TNBC cells through ATG5. Flubendazole has been reported to promote autophagy by activating the Atg4B that participates in autophagosome formation [22]. Hence, whether EVA1A promotes autophagic death of TNBC cells by activating Atg4B needs to be elucidated.

It has been reported that the mitochondria is involved in cell proliferation and death [23,24,25], suggesting that the mitochondria may be involved in the protective role of EVA1A against breast cancer. Mitochondria are important organelles in mammalian bioenergy, biosynthesis, cell homeostasis and signal transduction [26,27]. Yongqi Zhen et al., found that in MDA-MB-231 and MCF-7 cells, flubendazole increased the rate of mitochondrial permeability transition pore (mPTP) opening, decreased mitochondrial membrane potential and promoted the release of Cyto C from mitochondria into the cytosol, indicating that flubendazole damaged the permeability of the outer membrane of mitochondria in breast cancer. Flubendazole also induced mitochondrial dysfunction by decreasing ATP levels and upregulating superoxide dismutase in MDA-MB-231 and MCF-7 cells. The in-depth study showed that flubendazole promoted mitophagy by upregulating the expression of LC3, PINK1, Parkin and p-Parkinser65, and induced the translocation of Parkin to mitochondria in MDA-MB-231 and MCF-7 cells. Flubendazole upregulated dynamin-related protein (DRP1) and p-DRP1Ser616 in MDA-MB-231 and MCF-7 cells, and the inhibition of DRP1 with shRNA or inhibitor mdivi-1 significantly reduced the expression of PINK1 and Parkin promoted by flubendazole, indicating that DRP1 mediates flubendazole-promoted mitophagy in breast cancer. Moreover, the inhibition of mitophagy with *DRP1* shRNA mitigated flubendazole-induced mitochondrial dysfunction, indicating that flubendazole causes mitochondrial dysfunction by promoting DRP1-mediated mitophagy in breast cancer. The inhibition of mitophagy by *DRP1* shRNA or *Parkin* shRNA promoted flubendazole-inhibited cell viability and colony formation of MDA-MB-231 and MCF-7 cells, indicating that flubendazole suppresses breast cancer cell proliferation via promoting mitophagy. Furthermore, *DRP1*-knockdown also attenuated flubendazole-induced downregulation of MMP-2 and upregulation of E-cadherin, indicating that flubendazole suppresses breast cancer cell migration via promoting mitophagy. EVA1A siRNA partially inhibited flubendazole-induced DRP1 expression, decreased the expression of PINK1, Parkin and p-Parkinser65, and alleviated the decrease in mitochondrial membrane potential and ATP loss induced by flubendazole in MDA-MB-231 and MCF-7 cells, suggesting that flubendazole promotes DRP1-mediated mitophagy and mitochondrial dysfunction in breast cancer by upregulating EVA1A. EVA1A overexpression promoted DRP1-mediated mitophagy and inhibited breast cancer. Collectively, flubendazole suppressed breast cancer by inducing mitochondrial dysfunction through promoting DRP1-mediated mitophagy via upregulating EVA1A, which provides a new mechanism for targeting EVA1A in the treatment of breast cancer [28]. Moderate mitophagy can degrade damaged mitochondria, promote tumor cells to quickly adapt to these adverse conditions and inhibit their death [29]. However, excessive mitophagy damages the stability of the mitochondrial microenvironment and leads to the death of tumor cells [30]. In the above study, EVA1A induces mitochondrial dysfunction by promoting mitophagy, and then inhibits breast cancer, which may be related to mitophagy-induced apoptosis and autophagic death. The mechanism of EVA1A regulating autophagy in breast cancer needs to be clarified.

## 3. The Role of EVA1A in Papillary Thyroid Cancer

Papillary thyroid carcinoma (PTC), which is the most common kind of differentiated thyroid malignancy and accounts for 80% of all cases, is regarded as the least invasive type of thyroid cancer [31,32]. Its 10-year recurrence-free survival rate is about 95.5% [33]. Despite the good prognosis of most PTC patients after the treatment of surgical resection combined with radioactive iodine, the incidence rate and mortality of advanced PTC have significantly increased in recent years [34,35]. Therefore, it is particularly important to study the pathological mechanism of PTC and find new and effective treatment methods. Bang-Yi Lin et al., found that the expression levels of EVA1A in tumor tissues from 43 patients with PTC notably increased compared with that of adjacent normal tissues. Logistic regression analysis of the TCGA dataset demonstrated that EVA1A plays an important role in PTC. Silencing EVA1A by siRNA suppressed the proliferation, colony formation, migration and invasion of PTC cells. Silencing EVA1A by siRNA also induced apoptosis and suppressed epithelial-mesenchymal transition (EMT) progression evidenced by reducing the expression of N-cadherin, vimentin and Bcl-xL and upregulating Bax expression. In addition, Yes-associated protein (YAP) and transcriptional co-activator with PDZ-binding motif (TAZ), which are the two vital effectors of the Hippo pathway, were inhibited by EVA1A siRNA, indicating that EVA1A siRNA suppressed the Hippo pathway. Summarily, EVA1A could promote PTC progression and induced EMT by activating the Hippo pathway, although this needs further confirmation [36]. It can be seen from the above that the inhibition of apoptosis plays an important role in the role of EVA1A in promoting PTC. On the contrary, in the previous studies, EVA1A was able to promote apoptosis. The reasons for the contradiction may be related to the type of cancer and need to be further clarified. Furthermore, whether autophagy is involved in the role of EVA1A in promoting PTC remains to be studied.

## 4. The Role of EVA1A in Non-Small Cell Lung Cancer Cells

Lung cancer is one of the most common cancers in humans and one of the main causes of cancer-related death in the world. There are about 2 million new cases worldwide every year, with a mortality rate of 20% [37]. Non-small cell lung cancer (NSCLC), with 15% of the 5-year survival rate, accounts for about 85% of total lung cancers [38,39]. At present, the effect of the existing treatment methods for NSCLC is very limited. Therefore, it is urgent to explore the pathogenesis of NSCLC and seek better treatment methods [40]. Hong Xie and colleagues found that EVA1A protein expression was notably upregulated in a dose-dependent manner in H1299 cells (human non-small cell lung cancer cells). EVA1A overexpression mediated by adenovirus vector substantially inhibited H1299 cells’ growth. The in-depth study revealed that adenovirus vector-mediated EVA1A overexpression promoted autophagy by inducing autophagosome formation in the H1299 cells, which was further confirmed by EVA1A siRNA downregulating autophagy in A549 cells. Adenovirus vector-mediated EVA1A overexpression also induced apoptosis of H1299 cells and A549 cells. Furthermore, EVA1A induced H1299 cell arrest at the G2/M phase by inhibiting cell mitotic progression, indicating that EVA1A overexpression inhibited H1299 cell growth and induced cell death perhaps through inducing cell cycle arrest at the G2/M phase. To summarize, EVA1A inhibited NSCLC through inducing apoptosis, autophagy and cell cycle arrest, leading to cell death [41]. There are many mechanisms of cell death, and the specific mechanisms may vary depending on the stimulation and cell environment. The manner of cell death varies according to the nature of stimulation and the specific situation of the cell environment. In addition, apoptosis and autophagy have both synergistic and mutually exclusive effects [42]. The relationship between autophagy and apoptosis regulated by EVA1A merits further study in the future.

## 5. The Role of EVA1A in Hepatocellular Carcinoma

Hepatocellular carcinoma (HCC) is the most common primary liver cancer and a leading cause of cancer death [43,44]. Many factors are associated with HCC, such as alcohol consumption, hepatitis B and C viruses and nonalcoholic fatty liver [45,46]. Due to the difficulty of early diagnosis, most patients with HCC are found to be in the advanced stage. The effect of hepatectomy or radiofrequency ablation is poor and the recurrence rate is high [47]. Recently, studies have proved that oxaliplatin-based chemotherapy is an effective method for the treatment of advanced HCC. However, the serious drug resistance has significantly dampened its efficacy [48]. Therefore, it is urgent to find a method to inhibit the resistance of oxaliplatin-based chemotherapy. MicroRNAs (miRNAs), small endogenous single-stranded RNAs, can inhibit the target gene by binding to the 3′-untranslated region (UTR) of the target genes [49]. MiR-125b is the first mammalian homologous gene of mirna-lin-4 found in Caenorhabditis elegans, which regulates many physiological and pathological processes [50]. It has been reported that MiR-125b plays a vital role in HCC [51]. However, the relationship between MiR-125b and the drug resistance of HCC is not clear. Wei Wei Ren et al., found that MiR-125b expression in oxaliplatin-resistant HCC tissues and cells was reduced. MiR-125b overexpression in sensitive cells dampened oxaliplatin resistance by suppressing cell proliferation, migration and EMT compared with oxaliplatin-resistant HCC cells, while anti-miR-125b neutralized the changes, suggesting that miR-125b suppressed oxaliplatin resistance. The luciferase reporter gene assay showed that miR-125b inhibited EVA1A expression by binding to the EVA1A 3′-UTR, indicating that EVA1A is a target gene downstream of miR-125b. Furthermore, EVA1A expression was upregulated in tissues of oxaliplatin-resistant HCC, and its ectopic expression induced autophagy and abolished miR-125b inhibition of the growth of oxaliplatin-resistant cells and xenografts. In addition, miR-125b abolished the upregulation of beclin-1 expression and LC3-II/LC3-1 ratio and the downregulation of p62 expression induced by EVA1A, suggesting that miR-125b suppresses EVA1A-induced autophagy. Collectively, miR-125b reversed oxaliplatin resistance through inhibiting EVA1A-mediated autophagy. EVA1A promoted oxaliplatin resistance of HCC by inducing autophagy [52]. Autophagy has been reported to promote cancer drug resistance [53], and also the strengthened drug sensitivity of cancer [54]. The above self-contradictory report may be related to the type of cancer or drug. Therefore, the mechanism of autophagy in the regulation of HCC drug resistance by EVA1A remains to be studied.

It can be concluded from the above that EVA1A can induce HCC drug resistance and promote tumor development. In contrast, EVA1A has also inhibited HCC. Jiejie Yang and colleagues found that EVA1A expression was notably downregulated in HCC tissues and HCC cells, and was correlated with poor clinical prognosis and the clinical stage of advanced TNM in patients with HCC. EVA1A overexpression suppressed the migration and invasion of HCC cells by inhibiting the EMT that was the marker of cell invasion and migration. The results of western blot analysis revealed that the levels of TP53, BAX and p21 were notably upregulated, whereas the BCL-2 level was significantly downregulated in EVA1A-overexpressing HCC cells, suggesting that EVA1A overexpression induced p53/BAX-mediated apoptosis, p53/p21-mediated cell cycle arrest, and TP53 was the downstream target of EVA1A inhibition of tumor. Silencing TP53 with siRNA dampened EVA1A-induced inhibition of cell death, cells migration and invasion. In summary, EVA1 inhibited HCC through suppressing cell proliferation, migration and invasion via increasing TP53 [55]. Autophagy has been reported to be involved in cell proliferation, migration and invasion [56,57,58,59]. Then, whether autophagy mediates the improvement of EVA1A in HCC remains to be clarified in the future. In addition, the mechanism of EVA1A regulating TP53 in HCC is also worth studying. Upregulating EVA1A will be a good treatment strategy for HCC.

## 6. The Role of EVA1A in Glioblastoma

Glioblastoma (GBM) is the most common primary brain tumor in adults, with an incidence rate of 3.19/100,000 [60,61]. At present, the treatment of GBM is mainly tumor removal surgery, followed by radiotherapy and chemotherapy. The average survival time of patients is only 1–2 years, and the long-term prognosis is not ideal [62]. Therefore, there is an urgent need to explore the pathogenesis of this disease and find an effective treatment. Autophagy has been reported to play an important role in GBM; however, the mechanism is not completely clear [63,64,65]. Xue Shen and colleagues transfected the recombinant adenovirus 5-EVA1A vector (ad5-EVA1A) into glioblastoma (GBM) cells to overexpress EVA1A in order to analyse its antitumor activity in vitro. The results revealed that EVA1A overexpression inhibited GBM cell proliferation. The in-depth study showed that EVA1A overexpression induced autophagy by promoting autophagosome formation, dampening autophagosome clearance, upregulating the levels of LC3B-II and downregulating the p62 level [66]. The evidence indicates that the mammalian target of the rapamycin (mTOR)/ribosomal protein S6 kinase B1 (RPS6KB1) pathway plays a vital role in autophagy [67]. EVA1A overexpression significantly downregulated the phosphorylation of mTOR and RPS6KB1, indicating that EVA1A overexpression suppresses the mTOR/RPS6KB1 pathway. MHY1485 could activate mTOR and inhibit lysosome fusion. It could upregulate the phosphorylation of mTOR and RPS6KB1 dampened by EVA1A overexpression, and neutralize the effects of EVA1A overexpression on LC3B-II/p62, indicating that EVA1A overexpression promotes autophagy via inhibiting the mTOR/RPS6KB1 pathway. Moreover, EVA1A overexpression induced apoptosis 48 h after Ad5-EVA1A transfection rather than earlier (24 h). In summary, EVA1A inhibited the proliferation of GBM cells through promoting autophagy via suppressing the mTOR/RPS6KB1 pathway, which needs to be further verified in vivo [61]. It has been reported that many signaling pathways are involved in autophagy, such as the AMPK/mTOR pathway [68] and the Hippo/Yes-associated protein (YAP) pathway [69]; therefore, in addition to the mTOR/RPS6KB1 pathway, whether EVA1A can regulate autophagy through other methods in GBM needs to be further studied. Furthermore, autophagic death and apoptotic death are the mechanism by which EVA1A inhibits GBM; however, how EVA1A regulates apoptosis and autophagy in GBM needs to be studied.

## 7. The Role of EVA1A in Pancreatic Cancer

Pancreatic cancer is a tumor with one of the worst prognoses, and the only possible cure is surgical resection. However, when the tumor is found, it is usually late. Only a few patients can be operated on, and the five-year survival rate is less than 10% [70,71,72]. Since the pathogenesis of pancreatic cancer is not completely clear, exploring its pathogenesis will help to find a new treatment for pancreatic cancer [73]. The results of Ming Tao et al., showed that EVA1A is colocalized with glucagon and not insulin in islet cells, indicating that EVA1A specifically existed in islet alpha cells of the normal pancreatic tissue, and may maintain the architecture and function of normal alpha cells. EVA1A is strongly expressed in chronic pancreatitis, moderately or weakly expressed in pancreatic acinar cell carcinoma, but is not expressed in normal pancreatic acinar cells. Furthermore, EVA1A was not expressed in the alpha cells of pancreatic ductal adenocarcinoma, mucinous cystadenoma, solid papillary tumor, intraductal papillary mucinous tumor and pancreatic neuroendocrine tumor. Summarily, EVA1A might regulate the function of alpha cells because it was only distributed in islets’ alpha cells of normal pancreatic tissue. The ectopic expression of EVA1A in pancreatic tumors might promote the occurrence and development of tumors, which needed further research [6]. The role and mechanism of EVA1A in pancreatic cancer needs further study. EVA1A may become a potential target for the diagnosis and treatment of pancreatic cancer.

## 8. Conclusions

**Figure 1:**
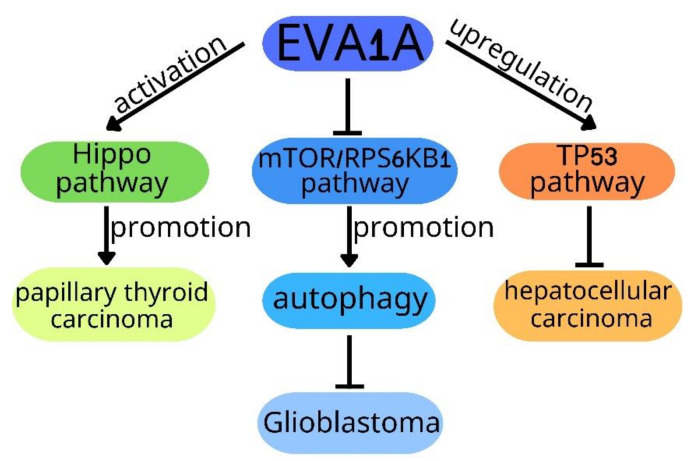
Signal pathways of the role of EVA1A in cancers.

In this review, we summarized the role of EVA1A in different types of cancers as follows: (1) EVA1A inhibits TNBC by inducing autophagic death of TNBC cells to inhibit tumor proliferation and migration; (2) flubendazole suppresses breast cancer through induction of mitochondrial dysfunction by promoting DRP1-mediated mitophagy via upregulating EVA1A; (3) EVA1A promotes PTC progression through activation of the Hippo pathway, which needs further confirmation; (4) EVA1A suppresses NSCLC by promoting apoptosis, autophagy and cell cycle arrest; (5) EVA1A enhances the oxaliplatin resistance of HCC through inducing autophagy; (6) EVA1A inhibits HCC by suppressing cell proliferation, migration and invasion via upregulating TP53; (7) EVA1A suppresses GBM cell proliferation by promoting autophagy via inhibiting the mTOR/RPS6KB1 pathway, which needs to be further verified in vivo; (8) the ectopic expression of EVA1A in the pancreas may contribute to the occurrence and development of pancreatic cancer, which needs further research (Table 1). It can be seen from the above that EVA1A mainly plays a role in cancer by regulating autophagy and apoptosis. Although previous studies have shown that EVA1A regulates autophagy through ATG5 and mTOR/RPS6KB1 pathways in cancer, the mechanism of EVA1A regulating autophagy in cancer is not completely clear and needs to be further clarified. This is equally the case for the mechanism of EVA1A regulating apoptosis. In addition, several signaling pathways are involved in the role of EVA1A in tumors, including the Hippo pathway, the mTOR/RPS6KB1 pathway and the TP53 pathway (Figure 1). Whether EVA1A plays a role in tumors through other signaling pathways remains to be clarified.

EVA1A sometimes promotes the development of cancer, and sometimes the contrary. The reason may be related to the type and development period of cancer, which needs to be further studied. Furthermore, some of the existing studies on the role of EVA1A in cancer only used in vitro experiments, so the results are relatively vulnerable and need to be further verified by in vivo experiments in the future.

The previous studies have shown that EVA1A suppresses NLRP3 activation to improve liver ischemia-reperfusion injury through induction of autophagy in Kupffer cells [74]. Our previous studies have shown that NLRP3 inflammasome and autophagy play an important role in liver inflammation [69,75]. Therefore, whether EVA1A can regulate autophagy/NLRP3 to improve liver cancer is a topic worthy of study in the future.

It is believed that with the deepening of research, EVA1A will provide a new strategy for cancer treatment.

EVA1A promotes papillary thyroid carcinoma progression through activating the Hippo pathway. EVA1A inhibits glioblastoma cells proliferation by upregulating autophagy via suppressing the mTOR/RPS6KB1 pathway. EVA1A inhibits hepatocellular carcinoma through suppressing cell proliferation, migration and invasion by upregulating TP53.

## Figures and Tables

**Table 1:** Summary of the role of EVA1A in different cancers.

The Type of Cancer	The Role and Mechanism of EVA1A in Cancer	Experimental Model	Reference
triple-negative breast cancer (TNBC)	EVA1A improves TNBC through promotion of autophagic death of TNBC cells via ATG5	TNBC cells and xenograft mouse models	[11]
breast cancer	flubendazole suppressed breast cancer through induction of mitochondrial dysfunction by promoting DRP1-mediated mitophagy via upregulating EVA1A	MDA-MB-231 and MCF-7 cells	[28]
papillary thyroid carcinoma (PTC)	EVA1A promotes PTC progression through the activation of Hippo pathway	the tumor tissue of the patients with PTC and PTC cells	[36]
non-small cell lung cancer	EVA1A inhibits NSCLC through promotion of apoptosis, autophagy and cell cycle arrest	lung cancer cell lines H1299 cells	[41]
hepatocellular carcinoma (HCC)	EVA1A enhances oxaliplatin resistance of HCC through induction of autophagy	the tumor tissue from oxaliplatin-resistant HCC patients and HCC cells	[52]
HCC	EVA1 inhibits HCC through inhibition of cell proliferation, migration and invasion via increasing TP53	the tumor tissue from oxaliplatin-resistant HCC patients and HCC cells	[55]
Glioblastoma (GBM)	EVA1A suppresses GBM cell proliferation through promotion of autophagy by inhibiting mTOR/RPS6KB1 pathway	GBM cells	[61]
Pancreatic cancer	the ectopic expression of EVA1A in pancreas promotes the occurrence and development of pancreatic cancer	human normal and neoplastic pancreatic tissues	[6]

## Data Availability

Not applicable.
